# Identifying the effects of social media on health behavior: Data from a large-scale online experiment

**DOI:** 10.1016/j.dib.2015.09.049

**Published:** 2015-10-13

**Authors:** Jingwen Zhang, Devon Brackbill, Sijia Yang, Damon Centola

**Affiliations:** University of Pennsylvania, Annenberg School for Communication, Philadelphia, PA, USA

## Abstract

Sedentary lifestyle is an escalating epidemic. Little is known about whether or how social media can be used to design a cost-effective solution for sedentary lifestyle. In this article we describe the data from a randomized controlled trial (RCT) that evaluated two prominent strategies for conducting exercise interventions using elements of social media: motivational media campaigns and online peer networks. The data file includes 217 participants’ basic demographic information, number of exercise class enrollments over 13 weeks, and self-reported number of days for exercise activities in the previous 7 days at baseline. Among the 217, 164 also have data on self-reported number of days for exercise activities at the post-program. Data are supplied with this article. The interpretation of these data can be found in the research article published by the authors in Preventive Medicine Reports in 2015 [1].

Specifications table

**Table:** 

Subject area	*Social Sciences*
More specific subject area	*Online social media and behavior change*
Type of data	*Excel file*
How data was acquired	*Digital records and survey from an online experiment platform*
Data format	*Raw*
Experimental factors	*Three experiment conditions: the control condition used a basic website; the media condition supplemented the control with professionally produced messages; the social condition supplemented the control with online peer networks.*
Experimental features	*Professionally produced media messages and online peer networks*
Data source location	*Philadelphia, Pennsylvania, USA*
Data accessibility	*Data is with this article*

**Value of the data**

• The data provides the first description of an RCT that tested the causal mechanism of an online social media intervention.
• The data contains both objective records of exercise class enrollment and self-reported survey measures on exercise activities.
• The data can be used to explore online intervention design and differences in the effects of media messages and online peer networks to increase physical activity.

## 1 Data

The data are from a 13-week social media-based physical activity promotion intervention called SHAPE-UP conducted at a Northeastern University in 2014.

## 2 Experimental design, materials and methods

### 2.1 The SHAPE-UP program as the research setting

SHAPE-UP was a 13-week exercise program that gave graduate and professional students at a large Northeastern university free access to an initial physical assessment and 49 exercise classes. Details of participant recruitment and contents of the exercise classes can be found in the original research article [1].

### 2.2 Description of the SHAPE-UP website and experiment conditions

The SHAPE-UP website was designed for participants to enroll in exercise classes and to interact with the program. To use the SHAPE-UP website, each participant created an anonymous online profile with an avatar. All participants had continuous access to the website. All exercise classes were pre-programmed in an online calendar. Upon clicking a class, participants could read a detailed description and register for it directly on the calendar. The registration then triggered a confirmation email sent to the participant immediately and a reminder email 12 h before the class started. In addition, an online tracking tool was built that participants could use to keep a daily journal of their class activities.

The basic SHAPE-UP website served as the control condition for the study. The website experiences across all three conditions were kept similar. The media condition added informational and motivational messages sent from the program and the social condition added social information on participants’ online peers. Example webpage illustrations for the three conditions can be found in the original research article [1].

**Fig. 1:**
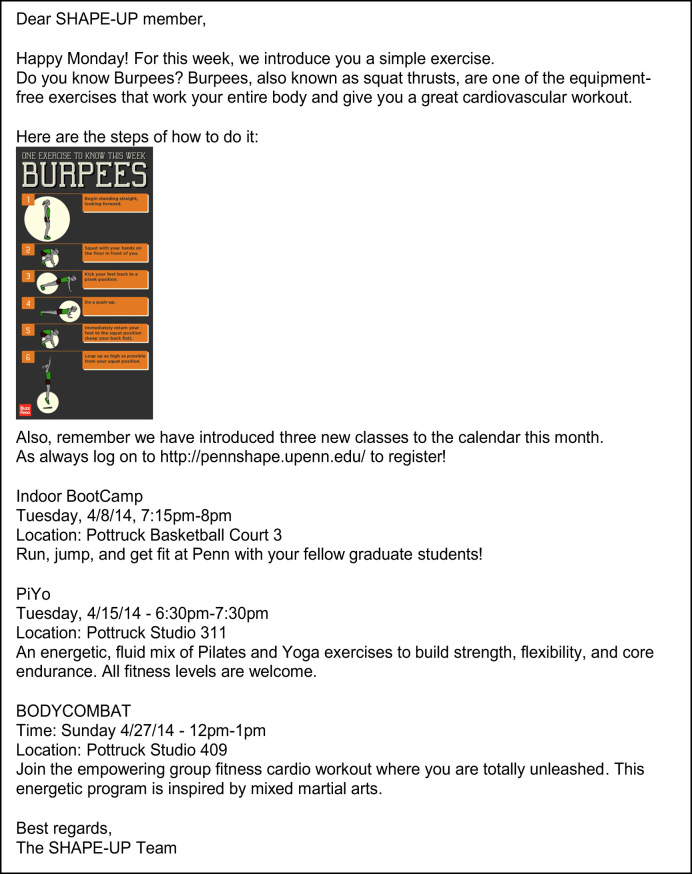
Example motivational email sent in the media condition.

In the media condition, participants received two videos on the SHAPE-UP website and one infographic that encourage physical activity on a weekly basis. As the first step, 38 videos were compiled from various physical activity campaigns. All videos were then coded on criteria including physiological arousal [2], [3], argument strength [4], production quality, and message sensation value [5]. A total of 24 videos coded as high on average across all criteria were used in the study. All branding information in the videos was cut out and edited by the authors to avoid confusion or emotional reactance to specific branding. For each week, participants received one video on Monday and one video on Wednesday, with real-time email notifications about these videos. Infographics were selected on a weekly basis from online sources including the health section of the New York Times, the social media site, Pinterest, and academic journals. Selected infographics were pre-evaluated qualitatively by all authors to be engaging, useful, and of high production quality. An example motivational email including one infographic is shown in Fig. 1.

**Fig. 2:**
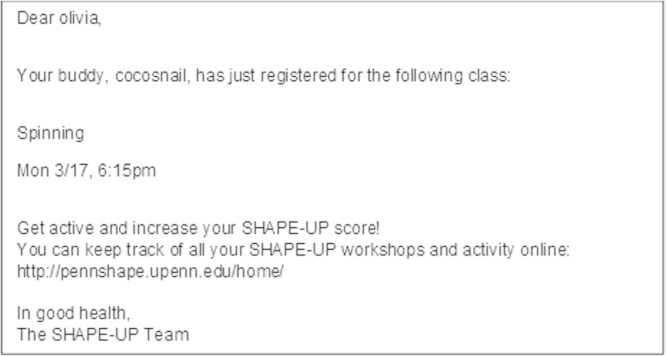
Example automated email on peers’ activities sent in the social condition.

In the social condition, participants were placed into online social networks, in which they were assigned four to six neighbors – referred to as “peers” – in the network. Participants could see peers’ profile information and class enrollment continuously updated in real time. Participants also received automated emails informing them when their peers enrolled in classes. An example automated email on peers’ activities is shown in Fig. 2.

## 3 Data collection methods

Participant enrollment and initial assessments were conducted from January 15, 2014 to February 1, 2014. Eligible participants completed a baseline online survey assessing demographic information and self-reported exercise activity. At the end of the program, one week after all the classes were finished, participants were contacted again for a post-program online survey.

The number of exercise class enrollments was recorded when participants digitally confirmed class registration on the SHAPE-UP website over 13 weeks.

Self-reported exercise activity was assessed at baseline and post-program through an online survey built into the SHAPE-UP website. The survey asked three questions developed by the Centers for Disease Control and Prevention [6]: number of days for moderate-cardiovascular (i.e., participating in physical activity for at least 30 min that did not make you sweat or breathe hard), intensive-cardiovascular (i.e., exercising or participating in physical activity for at least 20 min that made you sweat and breathe hard), and strength-building activities (i.e., doing exercises to strengthen or tone your muscles) in the previous 7 days. Responses ranged from 0 to 7 days.

Data collection was completed by May 2014. The data file includes basic demographic information, number of exercise class enrollments over 13 weeks, and self-reported number of days for exercise activities in the previous 7 days at baseline from 217 participants. Among the 217, 164 also have data on self-reported number of days for exercise activities at the post-program.
